# Monster, with a Head Resembling a Dog’s

**Published:** 1849-01

**Authors:** 


### ARTICLE IV.

### MONSTER WITH A HEAD RESEMBLING A DOG'S.

P. J. Hamilton, M. D., of Franklin, Wis., in a communication relates the following:—

On the 9th inst., I was called to see Mrs. R——, whom I found in labor with her fifth child. The pains were very strong when I arrived. The waters were discharged in a short time. I made an examination, and was at a loss to determine the presentation. The pains being strong, I was not long kept in suspense. A child was born with a dog's head. In every other respect, it was perfect. The cerebellum and part of the cerebrum were covered with their proper membrane; the dura mater. A cartilaginous substance formed part of the os frontis. The ears were long, and lopped down, like a dog's. The eyes were very large, and in every respect resembled a dog's. I learned the following particulars from the patient and her friends: When about six months advanced in her pregnancy, she went into a grocery store to purchase some articles. A couple of spaniel dogs got to fighting, and run against her, frightening her very much (she is of a nervous temperament). For several days after, she felt strong and unusual motions of the child. The fright of the dog was constantly occupying her mind, and the thought of her child being *marked*. The child weighed eight pounds.

The above case is one of hundreds that might be related where the resemblance, either real or supposed, of monstrous fœtuses to inferior animals or other objects can apparently be traced to some incidental influence during gestation.

An old and highly valued professional friend in Indiana, who is a firm believer in the agency of maternal mental influences in marking children, relates the following as coming under his own observation:—

A female acquaintance, during gestation, was greatly alarmed at the sight of her child, a son of several years age, who had been injured by the prong of a pitch-fork being thrust into his eye, so as to mangle it and cause the blood to flow in streams down his face. She was delivered, in due time, of a child, perfect in all respects, except that the corresponding eye to the one that had been injured in the boy was blood-shot, and a naevus maternus marked the course of the stream of blood down its cheeks.

Another.—A pregnant woman was greatly alarmed by her husband having thrown at her feet a squirrel that he had shot through the head so as to mangle it much. At her subsequent confinement, the child was found to have its head torn off very like the squirrel's.

A female, while pregnant, was badly frightened by going into a dark cellar, with a dim light, and observing in one corner a dog grinning and snarling at her. When the result of her gestation came forth, it proved that the superior maxilla protruded far in advance of the other features of the face, through a cleft in the upper lip, with two teeth standing out, and grinning like a dog. A friend of ours, at our elbow, who amputated the jaw, closed the hare lip, and related to us the story, has the bone yet.

A host of similar cases crowd upon our memory, but we have not room for more. If the cases of fright during gestation were summed up, we apprehend that there would not be one in a hundred where the child was not perfect. Therefore, as a cause of deformity, fright would seem to have but little influence. E.

